# Anti-Vaccine Discourse on Social Media: An Exploratory Audit of Negative Tweets about Vaccines and Their Posters

**DOI:** 10.3390/vaccines10122067

**Published:** 2022-12-01

**Authors:** An Nguyen, Daniel Catalan-Matamoros

**Affiliations:** 1 Centre for Science, Health and Data Communication Research, Bournemouth University, Bournemouth BH12 5BB, UK; 2 Medialab, Department of Communication and Media Studies, Madrid University Carlos III, 28005 Madrid, Spain

**Keywords:** vaccine hesitancy, anti-vaccination, fake news, misinformation, science controversy, anti-science

## Abstract

As the anti-vaccination movement is spreading around the world, this paper addresses the ever more urgent need for health professionals, communicators and policy-makers to grasp the nature of vaccine mis/disinformation on social media. A one-by-one coding of 4511 vaccine-related tweets posted from the UK in 2019 resulted in 334 anti-vaccine tweets. Our analysis shows that (a) anti-vaccine tweeters are quite active and widely networked users on their own; (b) anti-vaccine messages tend to focus on the “harmful” nature of vaccination, based mostly on personal experience, values and beliefs rather than hard facts; (c) anonymity does not make a difference to the types of posted anti-vaccine content, but does so in terms of the volume of such content. Communication initiatives against anti-vaccination should (a) work closely with technological platforms to tackle anonymous anti-vaccine tweets; (b) focus efforts on mis/disinformation in three major arears (in order of importance): the medical nature of vaccines, the belief that vaccination is a tool of manipulation and control for money and power, and the “freedom of health choice” discourse against mandatory vaccination; and (c) go beyond common factual measures—such as detecting, labelling or removing fake news—to address emotions induced by personal memories, values and beliefs.

## 1. Introduction

Before the COVID-19 pandemic, infectious disease decreasing ratios, which are reliant on a high vaccination uptake level, experienced a major global setback [1]. While there were many reasons for immunisation decline—such as vaccine shortages, lack of access, complacency towards disease risks, disinvestment in vaccine production—declining public confidence in vaccines emerged as the most critical problem [2]. This manifests in the rise of vaccine hesitancy—i.e., “delay in acceptance or refusal of vaccines despite availability of vaccination services” [3]—as a public health emergency all over the world. For example, as the world went in painful lockdowns to deal with COVID-19 and anxiously awaited a vaccine as the best solution to go out of it, many people still took to the streets of Western cities to call on others to oppose mandatory COVID-19 vaccination and, more generally, to stop sending children to immunisation. A survey in June 2020 [4] found that one in six UK adults “probably would not” or “definitely would not” get vaccinated when COVID-19 vaccines became available, with another sixth being unsure what they would do.

Although vaccine hesitancy is a “complex and context-specific” phenomenon that “(varies) across time, place and vaccines” [3], the rise of mis/disinformation about vaccination, fueled by the deep penetration of social media into daily life, has been defined as a key contributor. The COVID-19 pandemic, for instance, has been exacerbated by what the WHO [5] calls an “infodemic” from the outset, with its vaccines now struggling to deal with the spread of inaccurate, misleading, even fabricated information on social media about their proven safety and effectiveness. One study finds that vaccine opposition on Twitter increased by 80% during the course of the pandemic [6]. One of the Ten Actions Toward Vaccination for All that came out from the first Global Vaccination Summit in September 2019, under the joint auspice of the WHO and the European Commission, was to equip media and communication professionals with the skills and knowledge to fight false or misleading information on social media, alongside other tasks such as effective delivery of transparent, reliable vaccine information to the public.

This paper’s objective is to contribute to that effort by providing key quantitative and qualitative insights into the nature of anti-vaccine discourse on Twitter and their implications for health communicators and related stakeholders in their combat with vaccine mis/disinformation. The paper analysed data prior to the unprecedented COVID-19 health crisis in order to capture a ‘normal’ scenario and minimize ‘noise’ caused by this global pandemic.

### Anti-Vaccination Movements on Social Media: A Brief Literature Review

For centuries since the beginning of modernisation, vaccination has eliminated or reduced the rates of many once-feared infectious diseases, rendering itself to the status of one of the most successful measures of public health to date and one of the greatest achievements in human history [7]. At the heart of this success has been effective immunization campaigns in which health professionals, journalists and other stakeholders work together to build public understanding of, trust in, and adherence to vaccination [8]. These campaigns, for the most part, disseminated evidence about vaccine effectiveness and safety to increase societal awareness and motivate lay people to adopt and follow vaccination programmes. However, vaccine campaigners have over the past decades increasingly realised that they had never taken into account the fact that people are emotional beings and, as such, are not always rational consumers of science information [9]. A turning point was the publication in the Lancet of a small, observational study by Andrew Wakefield that falsely established a causal link between the MMR vaccine and autism [10]. Although subsequent exposure of the study’s methodological flaws and less than honourable motives led eventually to the retraction of the paper in 2010 and the removal of Wakefield from the British medical register, the massive media coverage that it received in those years sparked a public distrust in vaccines that has never gone away since then. In fact, Wakefield’s discredited research continues to serve as a key reference point for the anti-vaccine movement, with the disgraced doctor himself having become one of its celebrated faces, alongside names such as Donald J. Trump, Robert Kennedy Junior and Del Bigtree [11].

Under the influences of such scientific controversies around vaccines (i.e., Wakefield, 1999) and some individuals such as Trump, Kennedy Junior and Del Bigtree [11], and amidst the somewhat declining authority of scientific expertise in public debates [12,13], many parents—for reasons that have little to do with science and much with their life experience, political ideologies, religious beliefs, superstition and so on—are now less likely to accept research data and findings about vaccine safety and effectiveness [14]. Today, vaccine acceptance among parents is no longer a monolithic concept. As Leask et al. [15] found, societies now see five layers of parental positions about vaccines, which, in the order of prevalence, are the unquestioning acceptor, the cautious acceptor, the hesitant, the selective vaccinator (accepting some, not all, vaccines) and the refuser.

Such development has been fuelled in part by the fast and deep penetration of social media into daily life. Although social networking platforms have been used as an effective aid in spreading preventive messages against and increasing awareness of unhealthy behaviours [16], they have also emerged as a significant risk to vaccination. As content on social media does not need to undergo any editorial curation or scientific check, they foster a chaotic labyrinth of science, pseudo-science and non-science views. This, coupled with the global reach and immediacy of social media, has facilitated the staggering growth of harmful mis/disinformation—including an alarming amount of “fake news”, rumours and urban legends—about the benefits, safety and side effects of vaccines [17,18,19,20,21,22].

YouTube is a case in point. Basch et al. [23] analysed 87 vaccine videos with approximately 25,000 views on YouTube, the largest network for video sharing, to find that 65% of them broadcasted anti-vaccine message and 37% did not provide any supporting data. Another study [24] examined 35 YouTube clips about the human papilloma virus vaccine to find that 57% of them were anti-vaccine and that these were more likely to ignore scientific information or to describe issues inaccurately. The evidence, however, is still mixed. Covolo et al. [25] found that positive videos about vaccines on YouTube outnumber negative ones and are more viewed, but the latter were more liked and shared. This seems to have been the situation on other social platforms: Twitter and Facebook have been found to be home for far more pro- than anti-vaccine posts [26]. Gunaratne et al. [27] found that the decade leading to their research saw significant increases in the volume of pro-vaccine tweets and decreases in that of anti-vaccine tweets. However, despite the overwhelming volume of pro-vaccine content on social media, the size of mis/disinformed anti-vaccine communities online—and, by extension, in the offline world—has shown no sign of stopping and might be even growing. Soon after the first COVID-19 cases were reported, for instance, conspiracy theories—such as that the coronavirus was created by the pharma industry to sell a vaccine, or that Bill Gates created a microchip that would be implanted with the COVID-19 vaccine—began to spread on various social media platforms, especially Facebook, across the globe [28].

Such growth has often been attributed to another aspect of social media’s “dark power”: its ability to foster like-minded communities that are not always formed on the basis of rational evidence-based reasoning. According to this line of arguments, direct communication on social media between authors and readers allows and encourages users to search for not only factual information but also ideological confirmation and emotional support [29]. Many posts are therefore promoted thanks less to their factual value than to their social appeal, especially as it is manifested in the accumulation of “shares” and “likes” [30]. Researchers have observed that anti-vaccination posts on social media tend to stimulate more content interactions than pro-vaccine ones. On Instagram, for example, Basch and MacLean [31] found that anti-vaccine posts had a significant higher average number of likes. On Twitter, Blankenship [32] found that anti-vaccine tweets were four times more likely to be re-tweeted than neutral ones. On Facebook, Gandhi et al. [33] found from a large sample of vaccine content during 2015–2018 that anti-vaccine posts are more popular than pro-vaccine ones. Over time, it is argued, this results in each user developing an exclusive network of contents and connections with people having very similar beliefs and attitudes. When they are exposed the “right” sort of information—i.e., information that, regardless of truthfulness, confirms their biases and prejudices—many hardcore anti-vaccine individuals are likely to consolidate and spread their anti-vaccine stance to others through their online networks. In short, social media are believed to be the “right” platforms for anti-vaxxers to effectively receive, internalise and disseminate the “right” messages, however groundless and dangerous they might be. Such self-choice, supported by specific algorithms, allow users to be assigned to ideologically different sub-communities frequently identified as “echo chambers” [34].

Although the theoretical and empirical underpinnings of “echo-chamber” effects have recently been called in question in the area of political communication (e.g., [35,36]), there has been evidence of its existence in vaccine communication. Milani et al. [26], in a social network analysis of how vaccine-related visual content is shared on Twitter, found that while pro-vaccine gatekeepers (health professionals and NGOs for the most part) are fragmented in loose, strategic connections around new vaccine developments, anti-vaccine users (primarily parents and activists) interact frequently with each other to share views and strengthen their relationship. The latter, they observed, results in an abundance of reassuring content that consolidates and entrenches users’ beliefs against immunisation. Earlier, in their automatic meaning extraction of over three million tweets over a three-year period, Mitra et al. [37] arrived at the same conclusion: social media not only recruit new anti-vaxxers but also fortify their beliefs over time. In particular, they found that predisposition to general paranoia and distrust in government, when fuelled by relevant events on social media, is a key driver for many to join anti-vaccine movements. However, new anti-vaccine adoptees tend to be less certain and more social than long-established anti-vaccination users, who tend to be resolute and use closed in-group language to express and/or promote conspiratorial thinking and distrust in government. Similarly, Schmidt et al. [30], examining the evolution of the vaccine debate on Facebook through the liking and commenting behaviours of 2.6 million users over nearly 7.5 years, find that selective exposure leads over time to polarization of pro- and anti-vaccine groups. Further, like Milani et al. [26] and Gunaratne et al. [27], they found minimal cross-communication between pro- and anti-vaccine groups, with little effort on either side to bridge the divide. Other studies show that this sort of closed-group communication seems particularly likely to exist among conservative supporters who—as a result of more exposure to biased, misleading or deceptive vaccine information on social media—are more likely to believe in vaccine conspiracies, be concerned about vaccine safety and display a stronger vaccine hesitancy or [38,39,40,41]. More recently, Thelwall et al. [42] analysed 446 hesitant COVID-19 tweets to find a dominance of right-wing political opinions in anti-vaccine Twitter discourse.

However, there remains a gap in our understanding of the situation, namely the content-related characteristics of anti-vaccine posts on social media. While this might sound counter-intuitive in the context of the vast literature into anti-vaccine movements, the fact remains that there are few systematic studies into what types of anti-vaccine posts are more prominent and/or prevalent on social media. This is not to say that we know little about the common content of anti-vaccine messages being circulated on social media. We have known that, as Ward et al. [43] observe from an intensive literature review, such narratives often focus on three things: questioning the safety and effectiveness of vaccines, promoting personal freedom against mandatory vaccination, and showing distrust to governments, science, health professionals and the pharmaceutical industry. What seems to be missing in the literature, to the best of our knowledge, is attempts to break these three broad narratives into smaller categories to investigate the prevalence and, therefore, contribution of each category to the spread of vaccine mis/disinformation. After an exhaustive search, we found only two studies that focus exclusively on the content of anti-vaccine posts. One is a recent investigation of replying behaviours of Twitter accounts against COVID-19 vaccines by Miyazaki et al. [44], which finds that anti-vaccine tweeters reply the most to neutral users using toxic and emotional content. The other is an analysis by Love et al. [45] of 2580 vaccine-related tweets during a seven-day period, but this was just a brief research note with limited (and now dated) findings about the characteristics of anti-vaccine tweets. Further, there is a lack of available labels for many anti-vaccine content characteristics or behaviours, making it difficult to capture the key signals of misinformation [46].

As public health authorities, health communicators and the media hasten to work with technology firms to fight vaccine mis/disinformation [47], it is urgent for research to classify the characteristics of anti-vaccine messages into very specific categories as well as investigate how regularly users of social media are exposed to each category. Without a good grasp of these basic indicators, for example, it would be hard to know what to prioritise when designing a campaign to combat anti-vaccine on social media. Further, some promising content-based methods to tackle anti-vaccine mis/disinformation—e.g., the use of information inoculation tools such as the Fake News Game by Cambridge University [48]—will lack an empirical basis to be developed. This study will contribute new insights to this limited body of knowledge through an exploratory content analysis of anti-vaccine messages posted by UK residents on Twitter. It asks:

RQ1. What are the prevalent characteristics of anti-vaccine tweeters as reflected in their public profiles?

RQ2. What are the prevalent characteristics of anti-vaccine tweet content?

Another largely overlooked issue is the difference that anonymity might make to the content-related characteristics of anti-vaccine tweets. Anonymity has always been part of the attraction of the Internet as the feeling of being unknown to others can empower users and intensify their ability to speak out and express their personal views unadulterated [49], especially when it comes to sensitive or controversial topics such as anti-vaccination. It has been observed that sceptics who surf social media to advocate vaccine refusal have a significant anonymous presence on these platforms [20,50]. Writing under a mask affords users the full freedom to post content against common wisdom and/or mainstream knowledge accepted by authoritative groups such as scientists, medical professionals or public health officials. In addition, there has been a surge of bots and trolls masquerading as human users and trying to amplify anti-vaccine messages on social media. These automated users—characterized by their “three As”: activity, anonymity, and amplification—serve as an effective tool for anti-vaccine activists to spread their agenda and consolidate their arguments on social media [46,51]. Russian trolls and sophisticated bots, for example, publish vaccine tweets at considerably higher rates and volumes than a regular user [20]. To our knowledge, no analysis of social media discourse against vaccines has focused on identifying the characteristics and patterns of anonymous tweets or exploring how they might be different from non-anonymous ones. Therefore, we asked the following question:

RQ3. How do anonymous and non-anonymous anti-vaccine tweets differ from each other in terms of their content characteristics?

## 2. Materials and Methods

This study follows a quantitative design to analyse anti-vaccination tweets posted from the UK, a country that, as noted above, has recently seen an increase in vaccine scepticism and hesitancy. An exploratory content analysis, a strong and commonly used social research method for communication [52], was employed.

### 2.1. Sampling Procedure

Tweets about vaccines were collected using the Twitter advance search tool and the plug-in Ncapture with Chrome web browser and further analysed by the qualitative software NVivo 11 plus. NVivo allows downloading of tweets along with user-related information. Each tweet in the dataset is associated with the following information: full text, publication date, count of retweets, #hashtags used, other @users mentioned/addressed, poster’s declared location and poster’s coordinates (revealing the geolocation from where the tweet was posted), biography, count of tweets by the poster, count of tweeters following and being followed by the poster. Previous research has already used this sampling procedure [53,54].

All tweets containing two keywords—vaccin* and immunizat*—were searched and retrieved in real time between 4 March and 3 June 2019. Due to the sheer number of tweets, we followed previous research [55,56] to randomly select 15 different days of the sampling period to build a composite sample. This composite sample is a combination of individual days’ samples over the specified period. In total, we collected 266,396 tweets containing the keywords. From the initial sample, we excluded those that were not original tweets (i.e., retweets, *n* = 168,508), those whose coordinates were outside the UK (*n* = 92,799), those that were not in English (*n* = 11), those that were duplicates (*n* = 458) and those that used the term “vaccine” as a metaphor or spam (*n* = 109). The resulting sample included 4511 tweets, each single of which was then read and analysed for its tone (positive, neutral or negative). The final sample of 334 UK tweets (7.4%) that were negative towards vaccination was further analysed.

While this is a small sample, especially when compared to many previous automatic sentiment and other analyses of millions of tweets [57,58,59], it serves the purpose of an exploratory study undertaken to address the aforementioned gap in the literature. It should be noted that while software packages (e.g., NUD.IST, NVivo, QualPro) have made significant improvements for social scientists to automatically store and analyse data, the use of manual analysis is still important and it is being used in current research [32,60,61]. This is because there is no mechanistic replacement for complex processes of reading and interpretation and software does not provide “automatic” solutions to difficult problems and phenomena. Manual analysis of Twitter content prevents some of the disadvantages of computer-assisted analysis such as its simplistic coding and the Murphy’s Law [62,63], i.e., the very high likelihood of some possible computer and/or electronic application flaws will take place beneath the surface, without the knowledge or awareness of researchers who rely on their reliability [32,60,61].

### 2.2. Coding Process

We adopted an inductive coding approach for this study. We started with the raw NVivo dataset to identify the variables to be taken into our analysis. We arrived at two major groups: six background variables indicating the general Twitter profile of anti-vaccine posters (identities, locations, numbers of people followed by them, numbers of people following them, numbers of tweets they have posted) and eight content-related variables indicating the salient content aspects of the sampled tweets (type of content, used sources, primary frame, mentioned vaccine, number of used hashtags, number of users being mentioned, and number of retweets). The categories for each content variable, which we will detail in the Findings section, were first inductively coded from one subset of the sampled tweets and then deductively applied to the entire sample. All the sample was analysed by the authors with the initial support of a trained coder. To analyse the intercoder reliability level, we used Krippendorf’s Alpha to quantify the extent of agreement between raters for each variable on 30% of the final sample. The alpha values range between 0.87 and 1.00 and have an average at 0.92 across categories, showing a very high level of reliability.

Before presenting the findings, we should warn that many of the tweets quoted below are full of language problems, including spelling, grammar, syntax and expression errors, and even slang and swear words. This was a rather dilemmatic situation: we had to carefully consider whether to remove such problems from the tweets. In the end, we decided to keep them as raw as possible to reflect the very “rough”, sometimes uncivil nature of Twitter discourse. We did, however, intervene with light editing of tweets that were rather incomprehensible or had a high risk of being misunderstood. For ethical reasons, the analysis of findings will erase all mentions of individual users in quoted tweets, except when the mention is to direct a public figure (e.g., Matt Hancock, the UK’s former Health Secretary) or a public organisation.

## 3. Results

### 3.1. A Profile of Anti-Vaccine Tweeters

To answer RQ1, we explored the general Twitter profile of those posting anti-vaccine tweets (Table 1). As can be seen, the 334 tweets in our sample were generated by a total of 196 users, the vast majority of whom were posting from the UK (87%) and the rest from three British Crown Dependencies (Isle of Man, Guernsey and Gibraltar). In terms of identities, which were examined through the information provided in “user name” and “biography” texts, 48.5% of these unique users were classified as anonymous. In other words, almost half of the anti-vaccine tweeters in this sample did not provide enough data for others to identify them (e.g., no full name, nick/pseudo name, no occupation, no clear face picture, no affiliation, vague biography). Of those who did (i.e., non-anonymous users), the vast majority were ordinary lay people posting as parents and activists (39% of the sample), followed by the so-called “alternative health” practitioners (e.g., natural therapists, holistic therapists, homeopaths), celebrities, health professionals and experts, the media and so on.

Posters of the sampled anti-vaccine tweets are relatively active users: only 18% had tweeted less than 1000 times while more than half (52%) had made at least 5000 tweets. The median number of tweets made by this sample of users was 5015—i.e., 50% of the anti-vaccine tweeters had contributed at least 5015 posts to the Twitter sphere. However, the top 10% accounted for 62% of all the sampled anti-vaccine tweets. In terms of network sizes, anti-vaccine tweeters in the sample were followed by a median of 385 other users and followed a media of 454.

Prevalent content characteristics of anti-vaccine tweets. To identify the general characteristics of negative tweets about vaccines, we focused on eight specific variables, presented in Table 2. About 60% of the tweets (201) had an anonymous author. Of the tweets with identifiable authors (*n* = 131), 83% were by lay people (i.e., those with no specified health or health-related expertise), 5% by those self-identified as alternative health practitioners (e.g., homeopaths, natural/holistic therapists), 3% by scientists or health professionals, and 3% by the mainstream media.

In relation to the type of content, the majority of tweets (70%, *n* = 236) were based on personal opinion, with one-fifth using second-hand news and information, 9% using personal experience with vaccination and only 1.5% making a point on the basis of research findings of sorts. The majority of anti-vaccine tweets (74%) were posted from a general perspective with no mention of any specific vaccine, with measles, MMR and HPV being the most oft-mentioned specific vaccines in the rest of the sample (6.3%, 5.4% and 4.8%, respectively).

As for the origin of tweeted information, the majority (57%) of the sample did not include a clear source. Of those with clear source, the general picture is one that lacks a strong presence of more authentic science information sources—such as research findings published by public authorities or academic journals (only 3%) or the mainstream media (10%). Meanwhile, less authentic sources—such as social media, anti-vaccine websites (e.g., vaccineimpact.com) and alternative health sources (e.g., greenmedinfo.com, newstarget.com, naturalnews.com)—were present in 27% of the sample.

In terms of framing, our inductive coding resulted in 12 categories. 5.4% of the tweets are “frameless” as they contain nothing but links to some anti-vaccine content. Among the rest, vaccine safety was the most prevalent, with three distinctive framing categories. The first group, accounting for 10.3% of the sample, consists of tweets framing vaccines as containers of unsafe, toxic chemical compounds. Some of these highlighting this as a general problem—e.g., “Vaccination isn’t the issue; however, what the vaccines contain is the BIG issue!”—while others list a range of “toxic components” such as mercury, thiomersal, heavy metals, xenobiotics, fetal cells, baby foreskins and carcinogens.

The other two safety-framing categories are tweets linking vaccination specifically to autism (7.3%) and those highlighting other side effects—often expressed as “vaccine injuries” or “adverse reactions”—such as deaths, brain damages, sterilisation, fever and so on (27.2%). One example of this is the tweet by a widely followed homeopath:

*New enquiry this morning. 7yr old boy. Met all milestones up until 3 yrs old. After vaccines lost all speech & autism symptoms started. Doctors denied any link between the vaccs; the regression with ‘there’s no evidence’. Adverse reaction not recorded. How many more? #vaxxed*

A further 10.6% questioned the effectiveness of, and by extension the need for, vaccination. This is either indirectly through a dramatic story like the above by the Sun and Scottish Sun, or directly (sometimes uncivilly) as seen in the following:

*I hear that, but [they’re] not for me. The fact [is that] we’re still providing vaccines for something that’s no longer a common factor! Plus the government know there’s chance it can cause disability. They payout a maximum of £120k for each case. It’s a big risk to take.*

*Every time I see a meme about anti-vaxxers... I’m reminded I’d be some kind of poster child for them unintentionally. Never been vaccinated and I lived (past) 18.*

*How is not vaccinating dangerous; it’s the non vaxxed that don’t carry the diseases and the vaxxed that do carry them, so who’s the twat(?) Try educating yourself before getting into name calling you ignorant asshole. Away, take your dog to play in traffic.*

Beyond the “problematic” medical effects of vaccines are a range of categories representing non-medical concerns. The most frequent of these are tweets framing vaccination as a non-health enterprise associated with conflicts of interests, influences and manipulation by the pharmaceutical industry, media, politicians, public authorities and clinicians (39 tweets, or 11.5% of the sample). A further breakdown of these tweets shows that the majority (23 out of 39, or 59%) referred to the financial interests and influences of “big pharma”. These take the simple form of a headline with link to the sourced story such as:

*Merck Ramps Up MMR Vaccine Production Amid Measles Hysteria*

or a leading question:

*Is Bought movie exposing the ugly truth behind vaccines, GMO’s and the pharma industry? #BigPharma #vaccination*

or, in most cases, a deliberate argument:

*Money money money they take and ban the natural plants from human consumption, just to harvest and cultivate them into a profitable pill or vaccination.*

*Here is the REAL importance of the HPV vaccine for Merck. If the public believes it is possible to “vaccinate for cancers” the sky is then limit for them. Lymphomas, Leukemia, Pancreatic Lung & Breast Cancers, Brain Tumors etc, etc...; #IDONOTCONSENT #VaxWoke #HealthFreedom*

Alongside these are tweets that thrash out conspiracy theories around vaccines (5.1%). In March 2019, for example, in response to Amazon’s decision to remove anti-vaccine documentaries from its Prime service (which it made to calm public and political pressures), a UK-based anonymous tweeter claimed, with no evidence but a link to an anti-vaccine American doctor’s tweet, that it was just part of Amazon’s attempt to enter the pharmacy market:

*#doctors & #politicians long in bed w/ #Vaccine manufacturers, harming people -- NOW #AMAZON NOW JOINS THE GANGBANG. #vaxwoke #vaxxed*

Some others (4.5%) sought to sow doubts on the science of vaccination by framing it as methodologically or ethically questionable, such as the following:

*If they vaccinate from birth there can be no comparison studies. It really is a scene from Brave New World. Soon humans will be created in test tubes, viviparous birth will be seen as irresponsible. I hope you are right and that those responsible will get their just desserts.*

This was followed by vaccination-as-choice tweets, which project obligatory vaccination as a violation of parental rights to choose what they see as best for their children (9.4%). Some examples include the following:

*I definitely, 100% do not support mandatory vaccination. There are risks of injury and death to every vaccine. Where there is risk, there must be choice. Additionally, you can’t force me to inject human diploid cells from aborted fetuses into my child.*

*Well said—the issue of #HealthFreedom isn’t about whether vaccines are effective or safe or both or whatever. The issue is about freedom, liberty, and being free to determine what goes in our own bodies. These children are a danger to no one.*

*Very soon this will be their argument for mandatory adult vaccination. Once they get the kiddies legislation done, they will say, “you’re right! We will NEVER have herd immunity until all the adults are forced vaxxed too. That one is coming.*

Next are tweets that framed anti-vaccine perspectives as being suppressed and censored by the mainstream elite. These make up 4.2% of the sample and can be exemplified by the following:

*Difficult to change when importance of vaccines are ‘sold’ to public on basis of necessity of max uptake bec of herd immunity & we hv stating nothing negative shld be communicated to public re vaccines!! Huge changes needed to improve public trust.*

*There are all manners of people against vaccines; not hard to find if you look. Most are simple parents of injured kids with no big platform. And the media moratorium on reporting the story in a balanced way is very suspicious. Any other topic, they would play up the conflict.*

*MASSIVE #censorship of #vaccine safety lack in full force. Thoughts?*

Finally, a small number of the sampled tweets (3.3%) contain nothing but a simple disp-quote or declaration of the poster’s anti-vaccine position in conversation with other tweeters, such as the following:

*Not sure why but I feel people think I’m pro vaccines. I’m not.*

*I’m not a supporter of vaccines. Why the hostility?*

*I haven’t vaccinated my child as was advised by her consultant at hospital, not to due to a condition she already has ⋯. i am not a child abuser.*

### 3.2. Anonymous vs. Non-Anonymous Anti-Vaccine Tweets

To answer RQ3 on the difference that anonymity makes to the content of tweets, we conducted a series of crosstabulation analyses with Chi-squared tests of independence, as presented in Table 3. Two original variables—the number of hashtags being used and the number of retweets—were binary-recoded due to the high number of cases with a zero value (i.e., cases with no hashtag or no retweet).

As can be seen, 29% of anonymous tweets, compared with only 7% of non-anonymous tweets, were from British Crown Dependencies (Chi2 = 23.5, *p* < 0.001). In other words, anonymous tweets are far more likely than non-anonymous (29% versus 7%) to have been posted from crown dependencies. It is quite striking to see, however, that all other tests show no statistically significant relationship between anonymous and non-anonymous tweets in terms of content types, frames, the number of mentions in tweets, the use of hashtags and the likelihood of being retweeted. In other words, the anonymity of posters does not make much state difference in the way they tweet negatively about vaccines.

## 4. Discussion

As an attempt to help detect health mis/disinformation on social media, this paper audits the key user and content characteristics of anti-vaccine discourse through an inductive content analysis of a three-month sample of UK tweets against vaccines. Before a further discussion, some limitations must be acknowledged. First, as the construction of communication content is rooted in a country’s culture [64], the results from this UK sample may not be entirely applicable to other countries. Future studies should examine tweets beyond the UK context—in English as well as non-English languages—to determine cultural and context specific characteristics. Second, our study examines only Twitter rather than the entire social media landscape. Although Twitter is widely used enough to provide some good indicators for further research, future studies will need to dissect the potential differences in the prevalence of various anti-vaccine frames and angles on different social media platforms. Third, our search strategy used keywords that refer directly to vaccines and might have missed relevant posts that may not use those keywords. For example, some vaccine opponents might try to get around social media censors by using terms like *va$$ine*. Fourth, although we started with hundreds of thousands of tweets, the final sample size of this study is rather small, especially in comparison with past research that uses computational methods to analyse massive numbers of tweets. As noted, this serves the purpose of an exploratory investigation, helping to shed light on some prevalent features of anti-vaccine tweets, identify some noteworthy trends that no automatic analysis can reveal, and provide a structured framework for further studies into social media content against vaccination. Nevertheless, the findings from this sample should be treated with a healthy level of skepticism until other large-scale analyses of anti-vaccine posts on social media are undertaken to confirm or reject them.

That said, however, the findings provide some important insights into anti-vaccine discourse on social media and what could be done to limit their effects:

First, in line with previous studies, we found that anti-vaccine posts constitute a very small part of vaccine discourse on Twitter, accounting for just over 7% of all tweets about vaccination during the study period. Further, the data show that most anti-vaccine tweet authors do not seem to interact greatly with each other. In particular, nearly a half of sampled tweets mentioned no other user and another 28% did one; 82% used no hashtags; and about 81% were not retweeted at all. This somewhat contradicts previous social network analyses (e.g., [26,37])and needs further investigation with larger samples over longer time periods to confirm and explain.

Second, tackling anti-vaccine discourse on social media should start from targeting the tiny proportion of hyperactive anti-vaccine users who are not only productive but also well connected. Our data show that the top 10% of anti-vaccine Twitter accounts produce most of the sampled tweets and have more following and followed users than the rest of UK tweeters. A more qualitative examination of top anti-vaccine tweeters in our sample shows that they include very influential people and organisations outside Twitter. Of the three most followed anti-vaccine Twitter accounts during our study period, two are sister news outlets, one with nearly 1.5 million (first on the list), and the other one with about 45,000 (third on the list). These outlets contributed few anti-vaccine posts to the Twittersphere but the sheer number of their followers can make any single tweet more impactful than a combination of many. In addition, five of the ten most followed anti-vaccine accounts in our sample were owned by famed people—namely a conspiracy theorist, writer and footballer (200,000 followers), a boxing champion (39,000), a best-selling thriller author (35,000), a MC/musician/producer (13,000) and a concept artist (10,000). While these users contributed only six of the 334 tweets in the dataset, it is likely that much more people were exposed to their tweets than others. As an indicator, although 81% of anti-vaccine tweets in our sample were not retweeted at all, the footballer’s two tweets were shared by 22 and 13 other users.

Third, health communicators, scientists and policy-makers will need to work closely with technological platforms to tackle anonymous anti-vaccine tweets. Despite their freedom to express under a mask, we found little evidence that anonymous tweeters are different from non-anonymous ones in terms of their content characteristics. It was quite striking, however, to find that anonymous users accounted for less than half of the tweeters in this sample (Table 1) but produced more than 60% of all the tweets (Table 3). This suggests that anonymous anti-vaccine users are more active and more productive than their counterpart in posting content. This might be due in part to the fact that many of these are bots and trolls that, as noted earlier, have been found to be significantly more productive and more likely to spread anti-vaccination content or to sow discord [20,46,51]. They need to be closely monitored, flagged up to users and, when necessary, removed from the system. In this light, recent toolkits and guidelines to combat vaccine mis/disinformation by authorities such as the WHO [65], UNICEF [66], ECDC—European Centre for Disease Control [67] and the UK Government [68]—as well as recent strategies taken by social platforms (e.g., Google, Facebook and Twitter) to close on and de-platform spreaders of misinformation and hate speech—could serve as possible templates or initial references for future actions.

Fourth, our data suggest several major aspects of vaccine controversies that health communication initiatives should be focused on. The top priority would be mis/disinformation around the medical nature of vaccines, namely its supposedly harmful side effects (framed in 45% of our sampled tweets), its ineffectiveness (11%) and even its scientific standards (5%). Next on the list would be the largely cynical belief, including many conspiracy theories, that vaccination is a tool of manipulation and control for money and power by economic, political or cultural elites, especially the pharmaceutical industries. Another important thing to handle is the so-called “freedom of health choice” discourse: about 10% of the sampled tweets are not against vaccines per se but, due to certain ideologies, are simply against any move to make vaccination mandatory for their children.

Fifth, in line with previous research, our data show that anti-vaccine discourse on Twitter—and by extension, social media in general—is deeply rooted in anything but scientific knowledge and reasoning. The majority of the anti-vaccine tweets use personal opinion and personal experience rather than solid facts and figures of some sorts. In addition, they tend to rely on no source or, at best, very questionable sources, and are often “sweeping” in the sense that they criticise vaccines in general rather than aim at any specific vaccine. Such reliance on values/beliefs presents a formidable challenge to the tackling of online anti-vaccine discourse. This is because, as research has found, value disposition and prior beliefs can make it too difficult for people to modify false perceptions [69,70]. In some cases, explicit attempts to correct false beliefs with scientific facts can even backfire, leading individuals to consolidate initial beliefs [71]. As Nguyen and Catalan [72] argue, social media are the catalyst rather than the cause here: while they make it easy for mis/disinformation to surface and engender ill-informed public debates and dangerous decisions, the root of the problem remains that many people are willing to believe in things that, by normal intellectual standards, are unmistakably unscientific or counterintuitive. This problem, they say, entails a variety of human factors that can cloud public reasoning and/or be skillfully exploited for political, economic and/or religious gains.

In more practical terms, as emotions induced by personal memories, values and beliefs often form the basis for many to dismiss vaccines, health communicators and related stakeholders will need to seek creative ways to speak directly to such emotions rather than just using the traditional mode of top-down and fact-filled communication. Efforts to tackle misleading vaccine discourse on social media must go beyond common measures—such as detecting, labelling or removing mis/disinformation or “pumping” scientific facts into the debate—to address those “human factors” behind such beliefs and attitudes. Here, the aforementioned five-layer typology of parental positions toward vaccines [15] might be useful to devise communication strategies to target different groups with the relevant kind of intellectual and emotional appeals. We envision that each group will require a different set of tools and techniques to suit, inter alia, its emotional intelligence level, trust in healthcare systems, health and science literacy, news/media literacy, and openness to other sides of the debate. Without such customised approaches, as recent anti-vaccine protests amid the COVID-19 pandemic tell us, mis/disinformation is likely to continue to shape, consolidate and bring anti-vaccine beliefs and attitudes into irrational actions, with grave implications for public safety and human lives.

## Figures and Tables

**Table 1:** Characteristics of anti-vaccine tweeters.

	Frequency (%)
**Tweeters’ identities (*n* = 196)**	
Anonymous	48.5
Lay people	39.3
Celebrities	2.6
Experts (scientists or health professionals)	2.0
Alternative health practitioners	4.1
Media	1.5
Others (company, political party, organization)	2.0
**Tweeters’ locations (*n* = 194)**	
UK	87.1
British Crown Dependencies	12.9
**Number of tweets made (*n* = 185, median = 5675)**	
0–999	17.8
1000–4999	29.7
5000–9999	13.5
10,000 or more	38.9
**Number of following tweeters (*n* = 185, median = 385)**	
Less than 100	23.8
100–499	34.0
500–999	16.8
1000 or more	25.4
**Number of tweeters followed (*n* = 185, median = 454)**	
Less than 100	17.3
100–499	34.6
500–999	22.2
1000 or more	25.9

**Table 2:** Content characteristics of anti-vaccine tweets.

**Posters’ Identity (*n* = 332)**	
**Anonymous**	60.5
Non-anonymous	39.5
**Content type (*n* = 332)**	
Personal opinion	69.0
Personal experience	9.3
Non-personal content (e.g., news, research)	21.7
**Source (*n* = 331)**	
None	57.4
Research reports/journals	3.3
Anti-vaccine sources	9.1
Alternative health outlets	8.2
Social media (including blogs)	11.1
Mainstream media	10.0
Other (satire media, books, civil organizations)	0.9
**Frame (*n* = 331)**	
Unsafe compounds	10.3
Autism link	7.3
Other vaccine injuries	27.2
Questionable effectiveness	10.6
Non-health interests	11.5
Vaccination as choice	9.4
Conspiracies	5.1
Questionable science	4.5
Silenced voices	4.2
Anti-vaccine position establishment	3.3
Links to anti-vaccine content	5.4
Others	1.2
**Specific vaccine mentioned (*n* = 334)**	
None	73.8
Measles	6.3
MMR	5.4
HPV	4.8
Others (e.g., flu, mumps, yellow fever, hepatitis B, meningitis)	9.5
**Number of mentioned users (*n* = 332, median = 1)**	
Zero	45.8
One	27.7
2–5	23.0
6–14	0.0
15–24	4.5
**Number of hashtags (*n* = 333, median = 0)**	
Zero	82.0
One	7.5
Two	4.8
3–6	5.7
**Number of retweets (*n* = 301, median = 0)**	
Zero	80.7
One	9.9
2–5	6.3
6 or more	3.0

**Table 3:** Differences in content characteristics of anonymous and non-anonymous anti-vaccine tweeters (cross-tabulation with chi-squared tests).

	Anonymous Tweets	Non-Anonymous Tweets	
**Location** **[*n* = 325, Chi-squared (1) = 23.5, *p* < 0.001]**			
British Crown Dependencies	29.1	7.0	
UK	70.9	93.0	
**Content type** **[*n* = 330, Chi-squared (2) = 1.02, *p* = 0.60]**			
Personal opinion	67.8	71.0	
Personal experience	8.4	9.9	
Non-personal content (e.g., news, research)	23.6	19.1	
**Source** **[*n* = 329, Chi-squared (6) = 7.03, *p* = 0.32]**			
None	57.6	57.3	
Research findings/journals	2.5	4.6	
Anti-vaccine sources	11.6	5.3	
Alternative health outlets	8.4	8.4	
Social media (including blogs)	9.1	13.7	
Mainstream media	10.6	9.2	
Other (satire media, books, civil organizations)	0.5	1.5	
**Frame** **[*n* = 329, Chi-squared (11) = 14.7, *p* = 0.20]**			
Unsafe compounds of vaccines	9.1	12.3	
Autism link	6.5	8.5	
Other vaccine injuries	24.1	31.5	
Questionable effectiveness	10.6	10.8	
Non-health influences	11.6	11.5	
Vaccination as choice	10.6	7.7	
Conspiracies	6.0	3.1	
Questionable science	5.5	3.1	
Anti-vaccine pride	4.5	1.5	
Silenced voices	3.5	5.4	
Links to anti-vaccine content	7.5	2.3	
Others	0.5	2.3	
**Number of mentioned users** **[*n* = 324, Chi-squared (3) = 1.66, *p* = 0.65]**			
Zero	46.0	42.9	
One	29.3	26.2	
2–5	20.2	26.2	
6 or more (max = 24)	4.6	4.7	
**Using hashtags** **[*n* = 332, Chi-squared = 9.29, *p* = 0.16]**			
Yes	81.6	82.4	
No	18.4	17.6	
**Being retweeted** **[*n* = 330, Chi-squared = 0.013, *p* = 0.91]**			
Yes	80.4	80.9	
No	19.6	19.1	

## Data Availability

Data is available by contacting the corresponding author upon reasonable request.
